# Efficacy of Banxia Xiexin decoction for chronic atrophic gastritis: A systematic review and meta-analysis

**DOI:** 10.1371/journal.pone.0241202

**Published:** 2020-10-27

**Authors:** Yang Cao, Yixin Zheng, Jingbin Niu, Chunmei Zhu, Decai Yang, Fen Rong, Guoping Liu

**Affiliations:** 1 School of Basic Medical Sciences, Shanghai University of Traditional Chinese Medicine, Shanghai, China; 2 School of Public Health, Shanghai University of Traditional Chinese Medicine, Shanghai, China; Bhagwan Mahvir College of Pharmacy, INDIA

## Abstract

**Background:**

Banxia Xiexin decoction (BXD), a classical formula of traditional Chinese medicine (TCM), has been wildly used for chronic atrophic gastritis (CAG) patients with the cold-heat complex syndrome in China, and achieved satisfied effects. However, the clinical effects of it remains unclear.

**Purpose:**

The purpose of this article is to evaluate the clinical efficacy and safety of BXD for CAG treatment.

**Methods:**

We searched seven electronic databases including Ovid, Embase, PubMed, Cochrane Library, Wan-fang database, VIP (Chinese Scientific Journals Database) and CNKI (China National Knowledge Infrastructure) from their inception to September 21, 2020. We used Jadad scale and Cochrane Collaboration’s risk of bias tool to make evaluation of methodological quality. Revman 5.3 statistical software was used for statistical processing to evaluate the clinical efficacy and safety of BXD.

**Results:**

26 randomized controlled trials (RCTs) totaling 1985 patients were identified for analysis. Meta-analysis showed that BXD treatment was more effective (RR 1.29; 95%CI 1.24, 1.35; P<0.00001) and safe (MD 0.33; 95%CI 0.18, 0.58; P = 0.0002) than Chinese patent medicine + western medicine. Furthermore, BXD had improvement on symptoms scores such as stomach distending pain, and belching. Besides, BXD was more effective in inhibiting Helicobacter Pylori (HP), improving HP-related inflammation, and relieving the degree of glandular atrophy, intestinal metaplasia (IM), and dysplasia of gastric mucosa (GM).

**Conclusions:**

The meta-analysis showed that BXD was more effective and safer for CAG patients than the control group. However, due to limitations of methodological quality and small sample size of the included studies, further standardized research of rigorous design should be needed.

## Introduction

Chronic atrophic gastritis (CAG), characterized by the disappearance of the normal glands after suffering repeated damage, with or without IM [1], is now considered a premalignant lesion of gastric cancer (GC) [2]. The global incidence of CAG is about 0–10.9% [3], and the prevalence rates are higher in China [4]. GC is still a major world problem ranking fifth for cancer incidence and third for cancer-related mortality [5]. But with good prognosis and timely treatment in the early stage, GC patients can reach 90%~95% of 5-year survival rate [6]. Thus, sufficient attention should be paid to the management of CAG. HP infection is now considered an infectious disease and eradication is recommended in most cases [7]. However, recent studies showed that with the increasing antibiotic resistance, poor compliance of patients, and adverse drug reactions [8,9], the eradication rate of HP has become a challenge and decreased to below 80%. Since the commonly used therapies remain unsatisfactory efficacy and safety so far, it is important to look for alternative therapies to reduce symptoms of CAG patients and improve their quality of life [10]. Many sufferers have put their concentrations on alternative treatments such as TCM [11]. The TCM doctors judge the patient 's syndrome according to their tongue manifestation, pulse palpation and specific symptoms before using the medicine. Syndrome is a nomenclature of TCM. Actually, the cold-heat complex syndrome, which has some symptoms (signs) such as stomach distending pain, belching, yellow tongue coating, is common in the CAG patients. BXD has been wildly used for CAG patients with the cold-heat complex syndrome in China and achieved good effects [12]. However, the clinical effects of it remains unclear. Therefore, a systematic review and meta-analysis was conducted to evaluate its efficacy and safety.

## Materials and methods

### Search strategy

We searched for publications in the following databases from their inception until September 21, 2020 without language restriction: Embase, PubMed, Ovid, Cochrane Library, Wan-fang database, VIP (Chinese Scientific Journals Database) and CNKI (China National Knowledge Infrastructure). The following general wording of the search terms were individually used or in combination: “chronic atrophic gastritis”, “atrophic gastritis”, “Banxia Xiexin”, “Banxiaxiexin”, “cold-heat complex syndrome”, “precancerous lesions of gastric cancer” and “randomized controlled trial”.

### Selection criteria

The initial screening of titles and abstracts was independently completed. According to the titles and abstracts, studies were excluded if they were animal studies, case reports, reviews, experience introductions, non-randomized controlled trials (RCTs). Intervention measures combined with other therapies in the studies were excluded. If no obvious exclusion information was found, the study was temporarily included, and considered at a second screening.

A second screening was performed as a full- text browse by two researchers separately. According to the full- text, the selected studies were strictly screened according to the inclusion criteria. Disagreements were resolved by discussing with a third investigator.

Studies meeting our selection criteria were included in this meta-analysis. 1) Patients were definitely diagnosed CAG by endoscopy and pathology. 2) All RCTs published before September 21, 2020 were included. 3) The age of all participants was above 18 years old. 4) The experiment group used BXD, and the control group used conventional western medicine treatment, Chinese patent medicine treatment, placebo or no interventions. 5) Treatment course was not less than 1 month. 6) The Jadad score was not less than 2.

### Data abstraction and quality assessment

Two researchers independently performed data extraction, including the first author, publication year, sample size, sex, age, course of disease, intervention, duration, outcome measures, follow-up, side effects and Jadad score. Disagreements were resolved after consulting with a third investigator. We used the Jadad scale and Cochrane Collaboration’s risk of bias tool to make evaluation of methodological quality. The quality of randomized controlled trials (RCTs) was identified by the modified Jadad scale with the score ranged from 0 to 7. The modified Jadad scale included the following domains: randomization, concealment of allocation, blinding and patient dropouts. We investigated risk of bias in included studies using the Cochrane Collaboration tool which addresses the following: sequence generation, allocation concealment, blinding, incomplete outcome data, selective outcome reporting, and other sources of bias.

### Data synthesis and analysis

Review Manager 5.3 software was used for this statistical analysis. We calculated the pooled risk ratio (RR) to assess dichotomous data, while the weighted mean difference (WMD) was used for continuous variable data, with both adopting 95% confidence intervals (CI). Heterogeneity was statistically assessed by using the χ2 test and inconsistency index statistic (I^2^). A model of random effect was conducted if substantial heterogeneity existed (I^2^ >50% or P<0.05). Conversely, fixed effect model or subgroup analysis was used. The Z-test was used for the overall effect. Pooled results were considered statistically significant for p< 0.05. The potential publication bias was analyzed using the funnel plot.

## Results

### Description of studies

**Fig 1:**
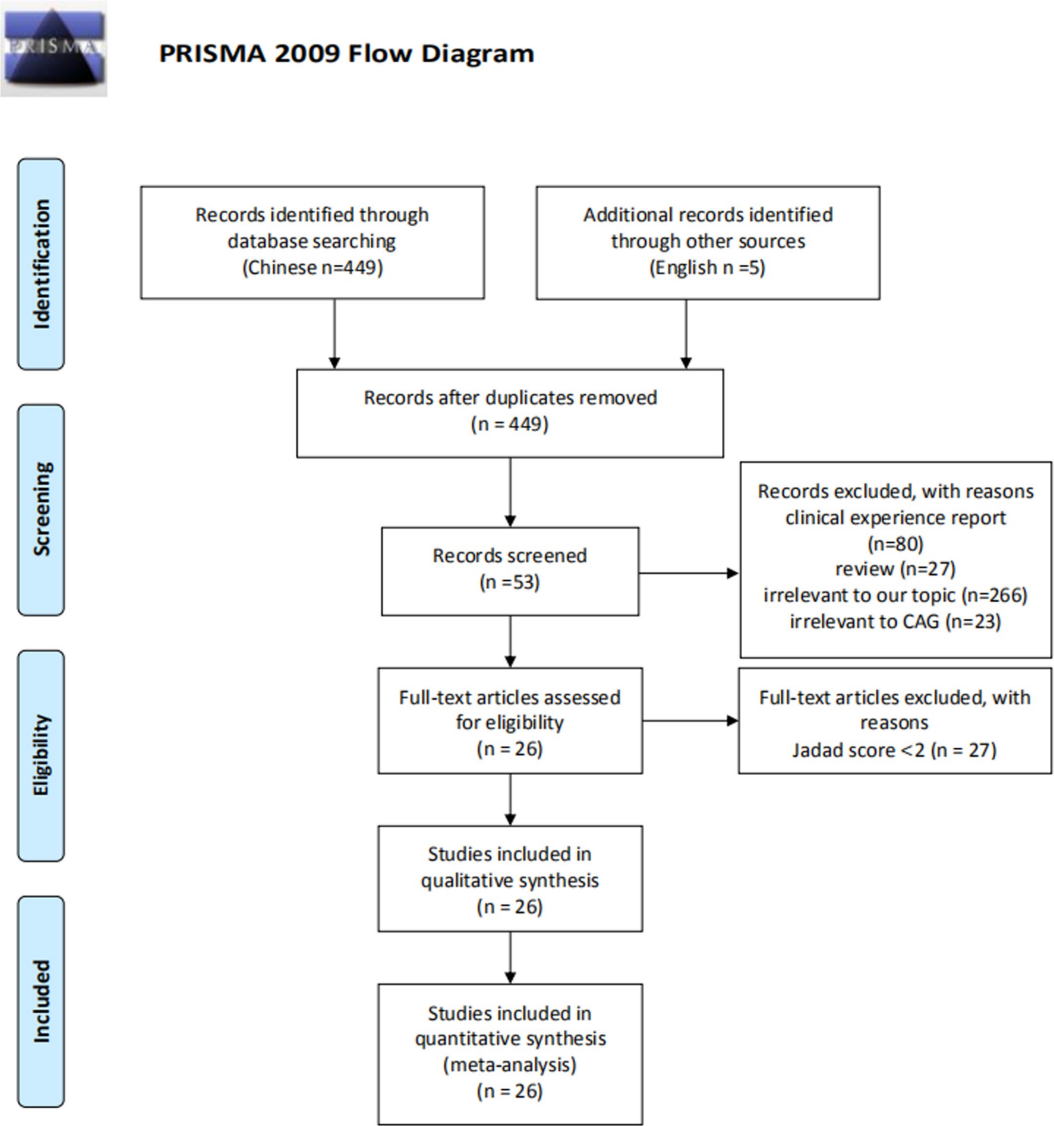
Flow diagram of included studies.

A total of 449 studies including 125 in CNKI, 214 in Wan-fang database, 110 in VIP, 2 in Cochrane Library, 1 in PubMed, 1 in Embase, 1 in Ovid were obtained based on the search strategy and screened records. 53 studies were included after the first screening. Articles with Jadad score less than 2 were further excluded after the second screening. 26 clinical RCTs satisfied our selection criteria were included in this meta-analysis [13–38]. The flow chart of literature search process was shown in Fig 1. Studies did not meet the inclusion criteria were excluded. Sample size was between 51 and 170. The ages of participants were from 18 to 81 years old. The course of treatment ranged from 8 to 24 weeks. The interventions between the experiment group and the control group was BXD versus western medicine + Chinese patent medicine. The characteristics of the included studies were described in Table 1.

**Table 1:** Main characteristics of all included studies in meta-analysis.

Study ID (First Author, Year)	Sample Size(E/C)	Intervention Measures			Course of treatment(days)	Methodological characteristics
		E	C	Final indicator		
Ling et al. 2018 (13)	54/54	**B**, 1 dose/d, b.i.d	999weitai, 1.25g, b.i.d	AD	90 days	Randomized Controlled
Tan et al.2018 (14)	35/35	**B**, 1 dose/d, 100ml, b.i.d	Pantoprazole Sodium Enteric-Coated Capsules, 40mg, q.d	AB	30 days	Randomized Controlled
Pei 2018 (15)	50/50	**B**, b.i.d	Omeprazole, 1 tablet, b.i.d, Amoxicillin, 2 tablets, b.i.d-t.i.d	EH	28–56 days	Randomized Controlled
Hu 2017 (16)	49/49	**B**, 1 dose/d, 100ml, b.i.d	Esomeprazole, 20mg, b.i.d, Hydrotalcite, 0.5g, t.i.d	DH	60 days	Randomized Controlled
Wen et al.2017 (17)	44/42	**B**, 1 dose/d, 200ml, b.i.d	Weifuchun, 1.44g, t.i.d	ADH	28 days	Randomized Controlled
Han 2016 (18)	40/40	**B**, 1 dose/d, b.i.d	Vatacoenayme tablets, 5 tablets, t.i.d	AE	90 days	Randomized Controlled
Zhong 2016 (19)	65/65	**B**,1 dose/d, b.i.d	lansoprazole tablets, 30mg, q.d	AE	60 days	Randomized Controlled
Hou et al.2016 (20)	38/38	**B**, 1 dose/d, 200ml, b.i.d	Weifuchun, 4 tablets, t.i.d	AE	28 days	Randomized Controlled
Li et al. 2016 (21)	40/40	**B**, 1 dose/d, b.i.d	Weifuchun, 4 tablets, t.i.d	ADE	30 days	Randomized Controlled
Zhang 2016 (22)	50/51	**B**, 1 dose/d, 150-200ml	Weifuchun, 1.44g, t.i.d	ABC	180 days	Randomized Controlled
Yang ZQ 2016 (23)	65/62	**B**, 1 dose/d, 200ml, b.i.d	Vatacoenayme tablets, 5tablets, t.i.d	AE	90 days	Randomized Controlled
Chang et al.2016 (26)	65/61	**B**, b.i.d	Vatacoenayme tablets, 5 tablets, t.i.d	ABE	30 days	Randomized Controlled
Yu 2015 (25)	50/50	**B**, 1 dose/d, b.i.d	Triple therapy(Omeprazole, 20mg, b.i.d; Amoxicillin, 0.5g, t.i.d; Clarithromycin, 0.25g, b.i.d)	A	30 days	Randomized Controlled
Ying 2015 (26)	26/26	**B**, 1 dose/d, 200ml, b.i.d	Weifuchun, 1.44g, t.i.d	ABCDH	28 days	Randomized Controlled
Liu et al.2014 (27)	60/60	**B**, 1 dose/d, 200ml, b.i.d	Weifuchun, 1.2g, t.i.d	ACD	84 days	Randomized Controlled
Qin 2013 (28)	40/40	**B**, t.i.d	Vatacoenayme tablets, 7 tablets, t.i.d+ Houtoujianweiling capsule, 4 tablets, t.i.d	AD	84 days	Randomized Controlled
Yang WT 2013 (29)	80/80	**B**, 1 dose/d, t.i.d	Vatacoenayme tablets, 7 tablets, t.i.d+ Houtoujianweiling capsule, 4 tablets, t.i.d	ADH	84 days	Randomized Controlled
Ke et al.2013 (30)	42/20	**B**, 1 dose/d, 150ml, b.i.d	Vatacoenayme tablets, 1g, t.i.d+ Weifuchun, 1.44g, t.i.d	ABCDH	90 days	Randomized Controlled
Wu 2013 (31)	28/28	**B**, 1 dose/d, 200ml, b.i.d	Domperidone, 10mg, t.i.d, Bismuth potassium citrate capsule, 110mg, q.i.d	ABDEH	120 days	Randomized Controlled
Wang 2012 (32)	30/30	**B**, 1 dose/d, b.i.d	Jianwei Xiaoyan granule, 20g, t.i.d	CDH	90 days	Randomized Controlled
Xu et al.2010 (33)	30/20	**B**, 150ml, 1 dose/d, b.i.d	Weifuchun, 1.436g, t.i.d	ABCE	90 days	Randomized Controlled
Yuan 2007 (34)	36/36	**B**, 1 dose/d, 200ml, b.i.d	Weifuchun, 4 tablets, t.i.d	ABEH	90 days	Randomized Controlled
Liu MX 2018 (35)	38/40	**B**, 1 dose/d, 175–200 ml, b.i.d	Quadruple therapy (Amoxicillin, 1g, t.i.d; Omeprazole, 20mg, t.i.d; Pectin bismuth capsule, 2 tablets, t.i.d; Clarithromycin, 0.5g, t.i.d)	ADEH	180 days	Randomized Controlled
Ma et al.2019 (36)	35/35	**B**, 200ml, 1 dose/d, q.d	Weifuchun, 4 tablets, t.i.d	ABE	N. R	Randomized Controlled
Wang.2019(37)	32/32	**B**, 1 dose/d, 200 ml, b.i.d	Weifuchun, 1.44g, t.i.d	ABC	84 days	Randomized Controlled
Wang.2020(38)	50/50	**B**, 1 dose/d, 200 ml, b.i.d	Weifuchun, 1.44g, t.i.d	ADEH	90 days	Randomized Controlled

Annotation: **B** = BXD; A = Clinical efficacy rate; B = Improvement of GM inflammation; C = Improvement in histopathologic changes of GM (glandular atrophy, IM and dysplasia); D = Improvement of symptom scores; E = HP inhibition rate; H = Adverse reactions; **E** = the experiment group; **C** = the control group; N. R = not reported.

### Risk of bias assessment

**Fig 2:**
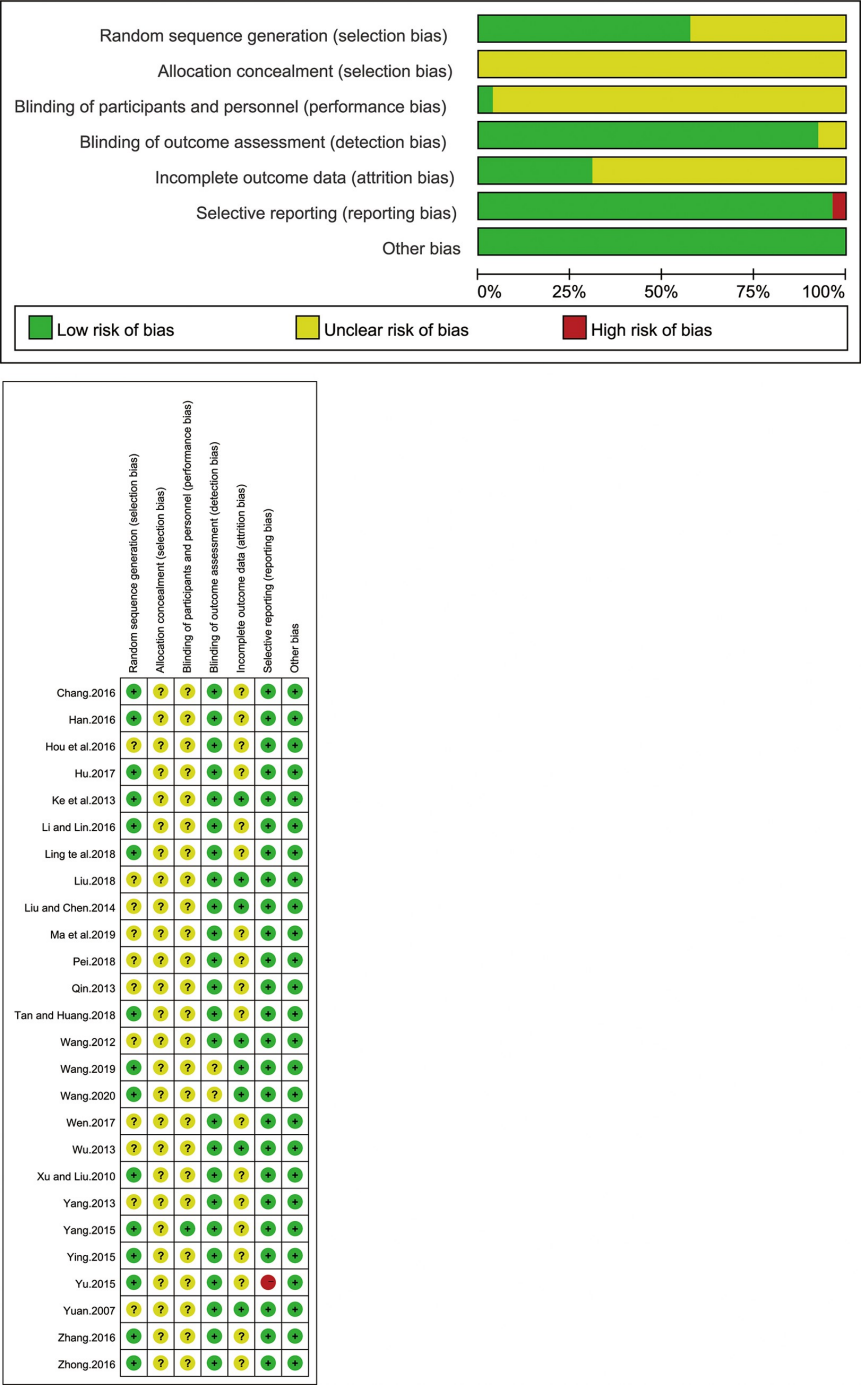
(a) Risk of bias graph. (b) Risk of bias summary.

We critically assessed the risk of bias of included studies according to the Cochrane Collaboration risk of bias tool. A description of the evaluation of methodological quality of the included studies can be observed in Table 2. 12 studies used random number tables [13,14,18,19,21,22,24–26,30,33,38]. Yang Zhongqiu [23] used method of flipping a coin. Hu Changning [16] used random colored balls method, Wang Nina [37] used method of randomly grouped by the computer, while the other 11 studies [15,17,20,27–29,31,32,34–36] used the word “randomization”, without any explanation of the random allocation process. The study of Yang Zhongqiu [23] was the only one reported “single blinded” method. The remaining 25 studies did not mention the blinding. None of included studies reported the allocation concealment. Six studies provided the number of dropouts [27,30,31,32,34,35], but did not conduct intention to treat (ITT) analysis for the missing data. Because of the relative lack of specific information, it cannot be determined whether implementations were conducted adequately in the process of random sequence generation, blinding or allocation concealment, thus accounting for the high risk in the validity of this review (Fig 2, (a) Percentage of risk bias, (b) Summary of risk bias). All studies [13–38] referred to the patients’ age, gender, etc. were comparable.

**Table 2:** Evaluation of methodological quality of all included studies.

Study ID (First Author, Year)	Baseline	Randomization	Allocation concealment	Blind method	Withdrawal or dropped-out	Follow-up	Side effects	Jadad score
Ke et al.2013 (30)	Comparability	Random number table	N. R	N. R	1 case dropped-out in the experiment group	N. R	no	4
Yang ZQ 2016 (23)	Comparability	Flipping a coin	N. R	Double-blind	N. R	N. R	N. R	3
Hu 2017 (16)	Comparability	Random color balls extraction	N. R	N. R	N. R	N. R	2 cases in the experiment group and 3 cases in the control group	3
Li et al. 2016 (21)	Comparability	Random number table	N. R	N. R	N. R	N. R	no	3
Ling et al. 2018 (13)	Comparability	Random number table	N. R	N. R	N. R	N. R	1 case in the experiment group and 4 cases in the control group	3
Liu et al.2014 (27)	Comparability	Random	N. R	N. R	3 cases dropped out in the experiment group and 5 cases dropped out in the control group	N. R	1 case in the experiment group and 2 cases in the control group	3
Liu MX 2018 (35)	Comparability	Random	N. R	N. R	7 cases dropped out in the experiment group and 5 cases dropped out in the control group	N. R	2 cases in the experiment group and 9 cases in the control group	3
Wang 2012 (32)	Comparability	Random	N. R	N. R	No cases dropped-out	N. R	no	3
Wu 2013 (31)	Comparability	Random	N. R	N. R	No cases dropped-out	N. R	no	3
Ying 2015 (26)	Comparability	Random number table	N. R	N. R	N. R	N. R	no	3
Yuan 2007 (34)	Comparability	Random	N. R	N. R	1 case dropped-out from the control group	N. R	no	3
Wang 2019(37)	Comparability	Randomly grouped by the computer	N. R	N. R	No cases dropped-out	N. R	1 case in the experiment group and 3 cases in the control group	3
Wang 2020(38)	Comparability	Random number table	N. R	N. R	No cases dropped-out	N. R	N. R	3
Chang et al.2016 (24)	Comparability	Random number table	N. R	N. R	N. R	N. R	N. R	2
Han 2016 (18)	Comparability	Random number table	N. R	N. R	N. R	N. R	N. R	2
Hou et al.2016 (20)	Comparability	Random	N. R	N. R	N. R	N. R	no	2
Pei 2018 (15)	Comparability	Random	N. R	N. R	N. R	N. R	4 cases in the experiment group and 14 cases in the control group	2
Han 2016 (18)	Comparability	Random number table	N. R	N. R	N. R	N. R	N. R	2
Hou et al.2016 (20)	Comparability	Random	N. R	N. R	N. R	N. R	no	2
Pei 2018 (15)	Comparability	Random	N. R	N. R	N. R	N. R	4 cases in the experiment group and 14 cases in the control group	2
Xu et al.2010 (33)	Comparability	Random number table	N. R	N. R	N. R	N. R	N. R	2
Yu 2015 (25)	Comparability	Random number table	N. R	N. R	N. R	N. R	N. R	2
Zhang 2016 (22)	Comparability	Random number table	N. R	N. R	N. R	N. R	N. R	2
Ma et al.2019 (36)	Comparability	Random	N. R	N. R	N. R	N. R	no	2
Yang WT 2013 (29)	Comparability	Random	N. R	N. R	N. R	N. R	no	2
Zhong 2016 (19)	Comparability	Random number table	N. R	N. R	N. R	N. R	N. R	2

Annotation: N.R = not reported.

### Outcomes

#### Comparison of clinical efficacy rate

**Fig 3:**
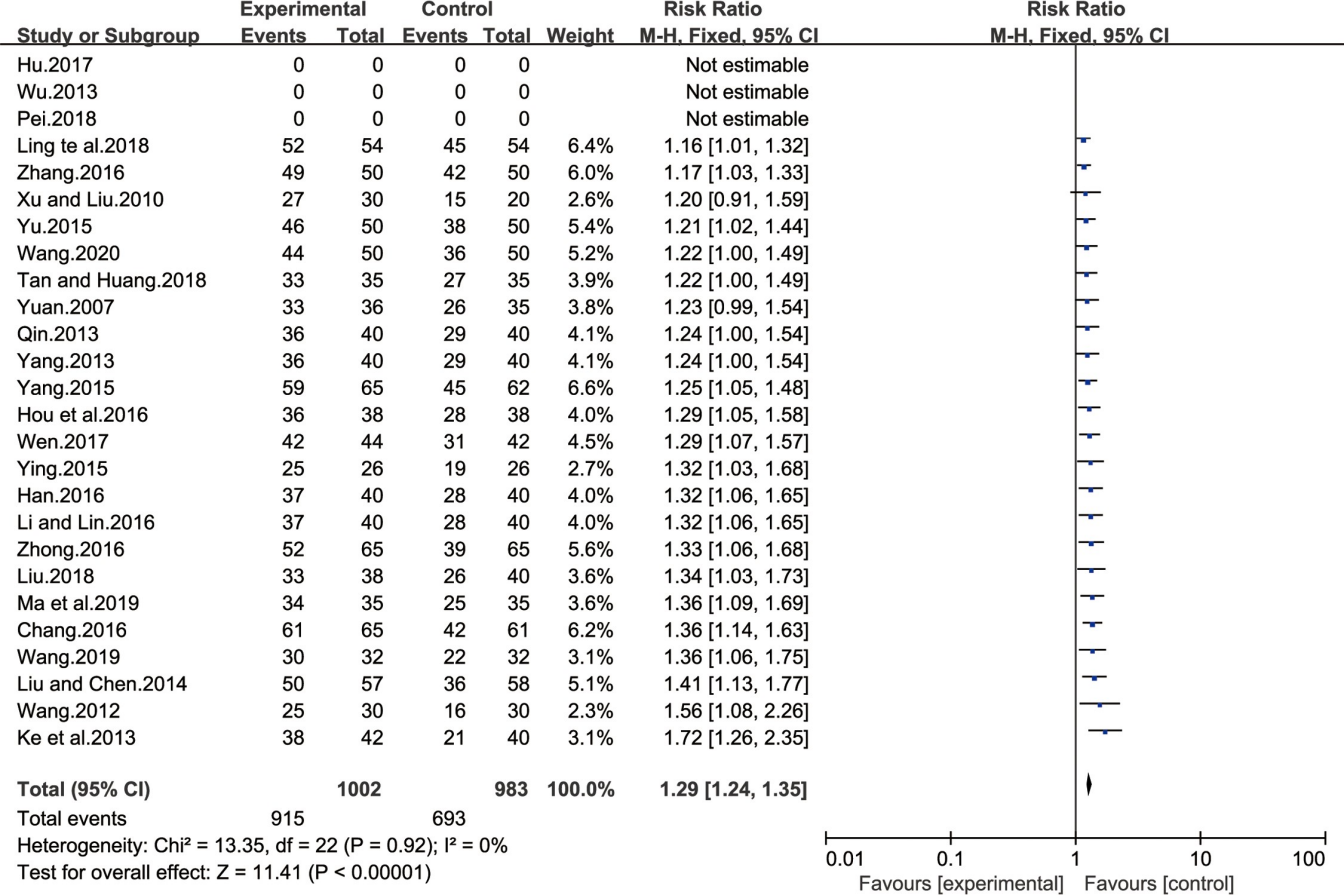
Forest plot of clinical efficacy rate (fixed effect model).

Among the included studies, twenty-three [13,14,17–30,32–38] reported the clinical efficacy rate. Heterogeneity analysis results (P = 0.92, I2 = 0%) showed homogeneity among 23 studies, so fixed effect model was used for analysis. BXD showed statistically significant differences in clinical efficacy rate compared to the control group (RR 1.29; 95% CI 1.24, 1.35; P<0.00001). The test for overall effects: Z = 11.41. It showed that clinical efficacy rate of BXD was better than the control group (Fig 3).

#### Publication bias

**Fig 4:**
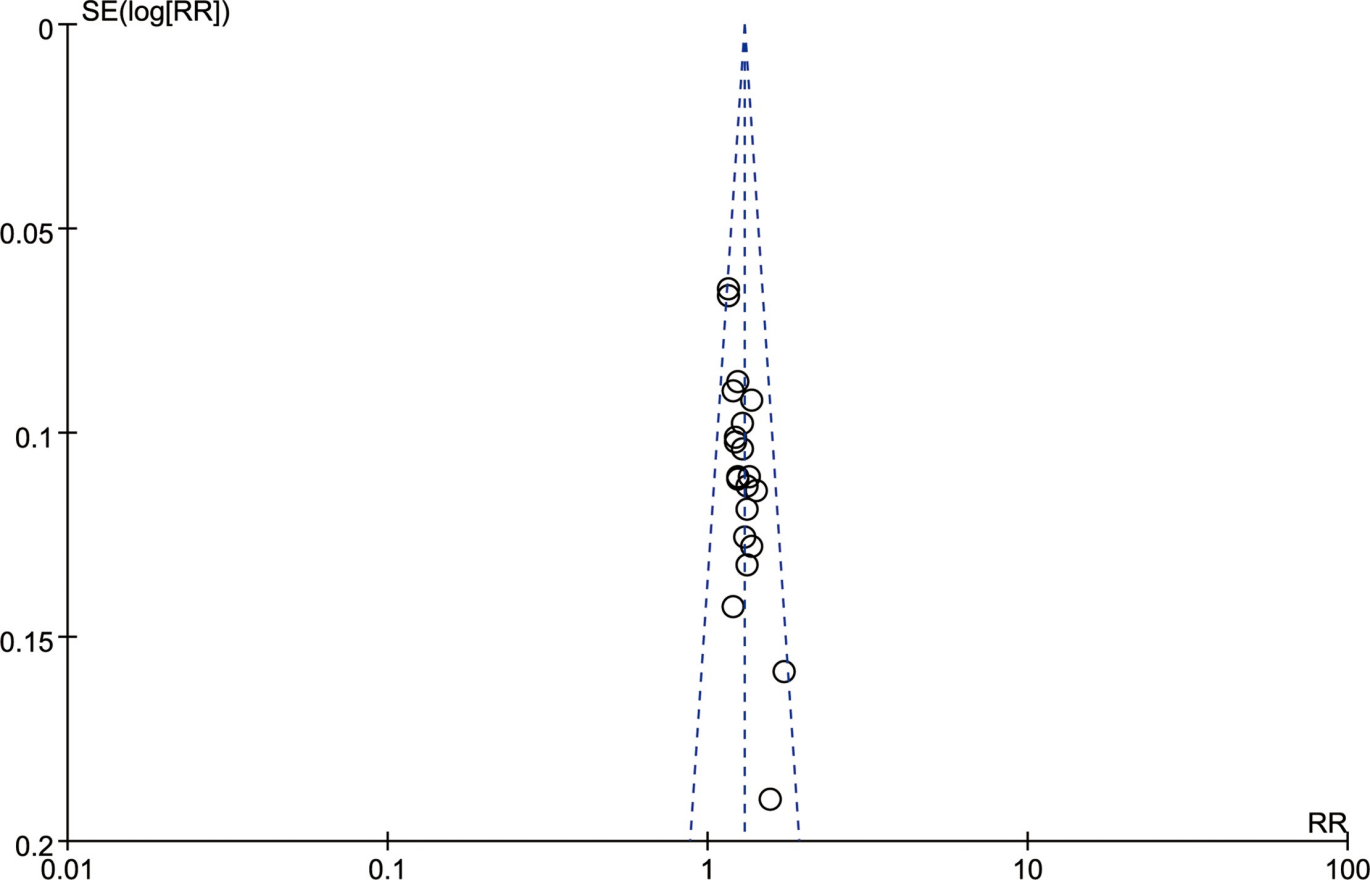
Funnel plot of effective rate.

Potential publication bias of the twenty-three studies [13,14,17–30,32–38] was identified by asymmetrical funnel plot (Fig 4).

#### Improvement of symptom scores

Twelve of all the included studies [13,16–17,21,26–29,31–32,35,38] reported the improvement of clinical symptoms. One study [27] only described the improvement rate of single symptom. Two studies [17,31] only described the change of overall symptom score without details. Due to the lack of information, it is impossible to conduct analysis and draw forest plot, so the three studies above were excluded.

**Fig 5:**
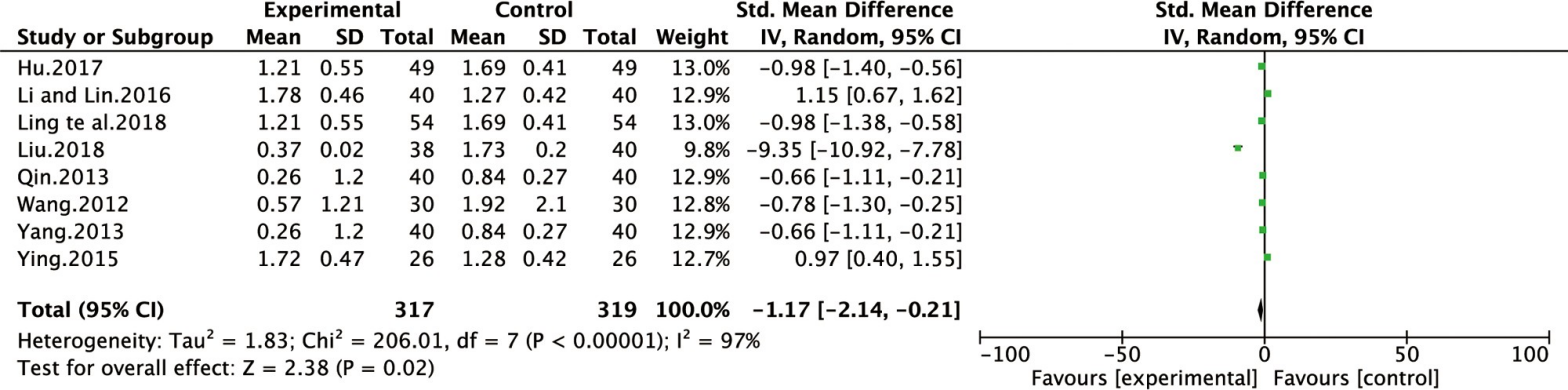
Forest plot of stomach distending pain symptom score (random effect model).

*Stomach distending pain symptom score*. Eight of all the included studies [13,16,21,26,28,29,32,35] reported the improvement of stomach distending pain symptom score. The heterogeneity test results were I^2^ = 97%, indicating that the study had heterogeneity, so the random effect model was adopted for analysis. BXD showed statistically significant differences in stomach distending pain symptom compared to the control group (WMD -1.17; 95% CI -2.14, -0.21; P = 0.02). The test for overall effects: Z = 2.38. The results showed that the stomach distending pain symptom in CAG patients with BXD was better than the control group (Fig 5).

**Fig 6:**
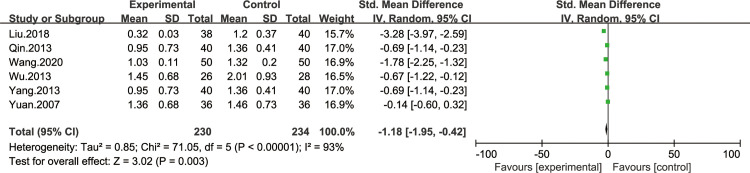
Forest plot of belching symptom score (random effect model).

*Belching symptom score*. Six of all the included studies [28,29,31,34,35,38] reported the improvement of belching symptom score. The heterogeneity test results were I^2^ = 93%, indicating that the study had a heterogeneity, so the random effect model was adopted for analysis. BXD showed statistically significant differences in belching symptom compared to the control group (WMD -1.18; 95% CI -1.95, -0.42; P = 0.003). The test for overall effects: Z = 3.02. The results showed that the belching symptom in CAG patients with BXD was better than the control group (Fig 6).

**Fig 7:**
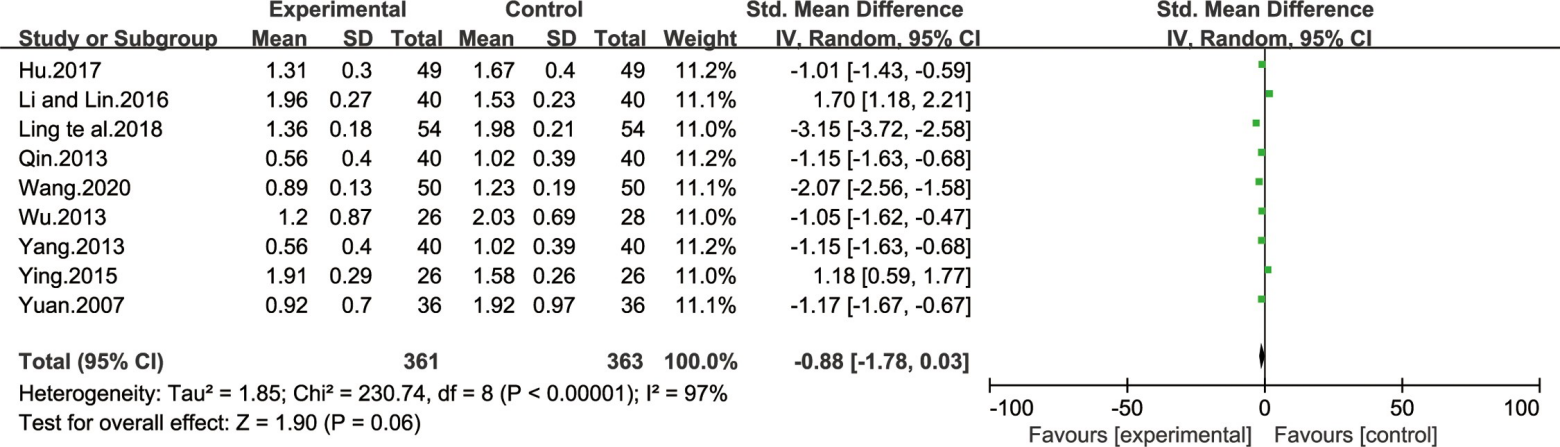
Forest plot of stomach fullness symptom score (random effect model).

*Stomach fullness symptom score*. Nine of all the included studies [13,16,21,26,28,29,31,34,38] reported improvement effect of stomach fullness symptom score. The result of heterogeneity test was I^2^ = 97%, indicating that the study had heterogeneity, so the random effect model was adopted for analysis. BXD showed no statistically significant differences in stomach fullness symptom compared to the control group (WMD -0.88; 95% CI -1.78, 0.03; P = 0.06). The test for overall effects: Z = 1.90. The results showed that the stomach fullness symptom in CAG patients with BXD was not better than the control group (Fig 7).

**Fig 8:**
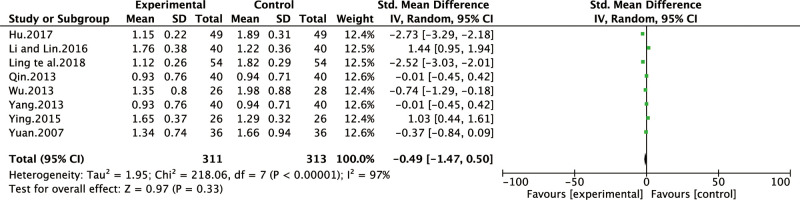
Forest plot of torpid intake symptom score (random effect model).

*Torpid intake symptom score*. Eight of all the included studies [13,16,21,26,28,29,31,34] reported the improvement of torpid intake symptom score. The heterogeneity test result was I^2^ = 97%, indicating that the study had a heterogeneity, so the random effect model was adopted for analysis. BXD showed no statistically significant differences in torpid intake symptom compared to the control group (WMD -0.49; 95% CI-1.47,0.50; P = 0.33). The test for overall effects: Z = 0.97. The torpid intake symptom in CAG patients with BXD was not better than the control group (Fig 8).

To sum up, the analysis of symptom results showed that BXD had advantages in improving the symptoms of stomach distending pain and belching, but BXD had no obvious effects on stomach fullness symptom and torpid intake symptom.

#### Improvement of GM inflammation

**Fig 9:**
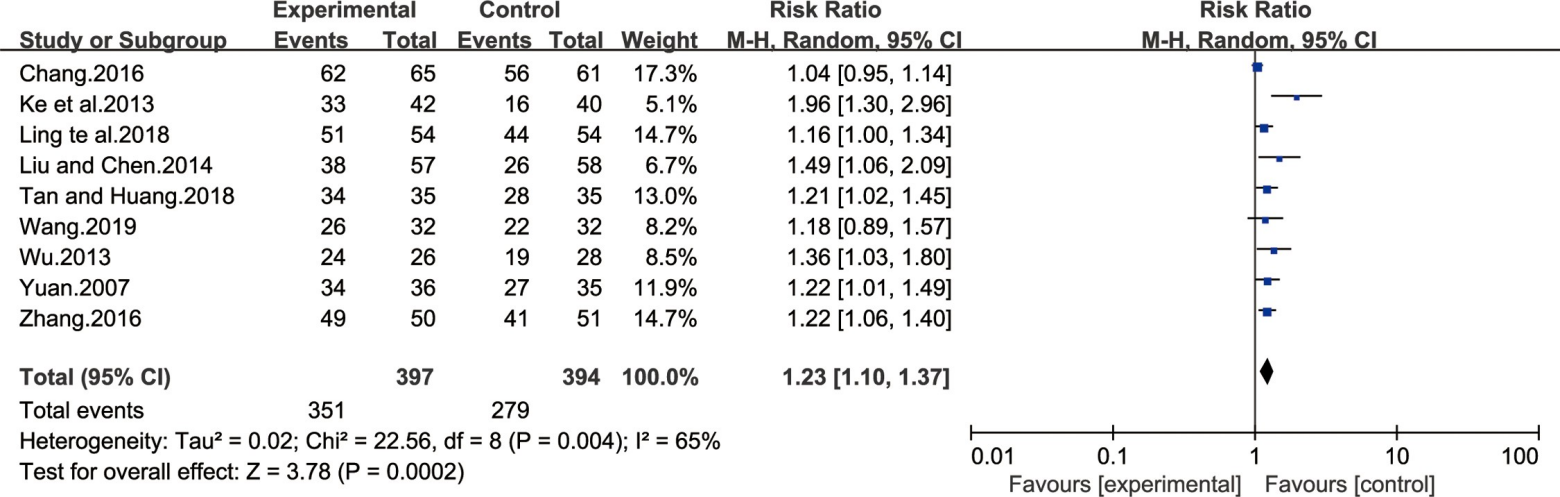
Forest plot of improvement of GM inflammation (random effect model).

Nine of all the included studies [13,14,22,24,27,30,31,34,37] reported improvement of GM inflammation on CAG patients after BXD treatment. The result of heterogeneity test was I^2^ = 65%, indicating that the study had a heterogeneity, so the random effect model was adopted for analysis. BXD showed statistically significant differences in improvement of GM inflammation compared to the control group (MD 1.23; 95% CI 1.10, 1.37; P = 0.0002). The test for overall effects: Z = 3.78. The results showed that the improvement of GM inflammation in CAG patients with BXD was better than the control group (Fig 9).

#### Improvement in histopathologic changes of GM (glandular atrophy, IM, dysplasia)

**Fig 10:**
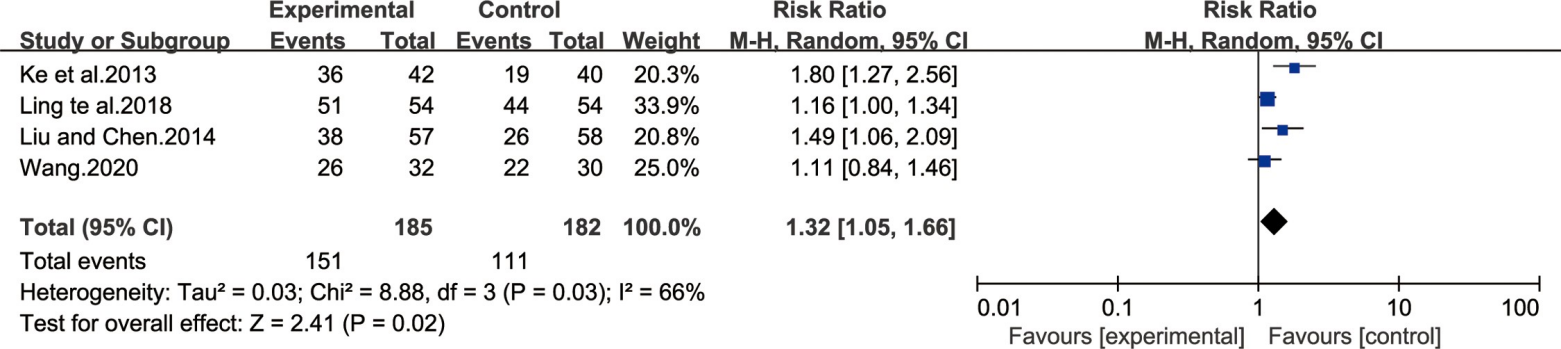
Forest plot of improvement in histopathological changes of GM (random effect model).

Four of all the included studies [13,27,30,38] reported the improvement in histopathologic changes of GM after treatment, including glandular atrophy, IM and dysplasia. The result of heterogeneity test was I^2^ = 66%, which showed that the study had a heterogeneity, so the random effect model was adopted for analysis. BXD showed statistically significant differences in the improvement of histopathologic changes of GM compared to the control group (MD 1.32; 95% CI 1.05, 1.66; P = 0.02). The test for overall effects: Z = 2.41. The results showed that histopathologic changes of GM in CAG patients with BXD was better than the control group (Fig 10).

#### HP inhibition rate

**Fig 11:**
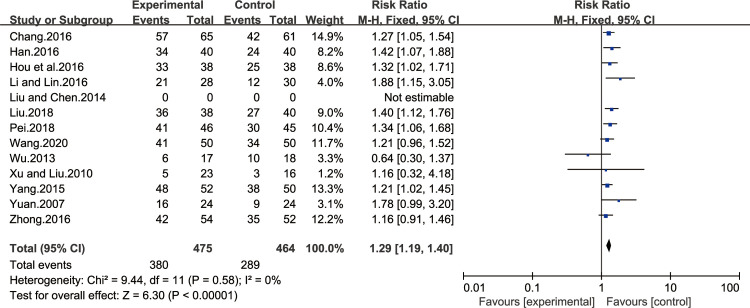
Forest plot of HP inhibition rate (fixed effect model).

Thirteen of all the included studies [15,18–21,23,24,27,31,33–35,37–38] reported the improvement of HP inhibition rate after treatment. The results of heterogeneity test were P = 0.58 and I^2^ = 0%, which showed that the study had a homogeneity, so the fixed effect model was adopted for analysis. BXD showed statistically significant differences in HP inhibition rate compared to the control group (MD 1.29; 95% CI 1.19, 1.40; P<0.00001). The test for overall effects: Z = 6.30. The results showed that the HP inhibition rate in CAG patients with BXD was better than the control group (Fig 11).

#### Safety evaluation

**Fig 12:**
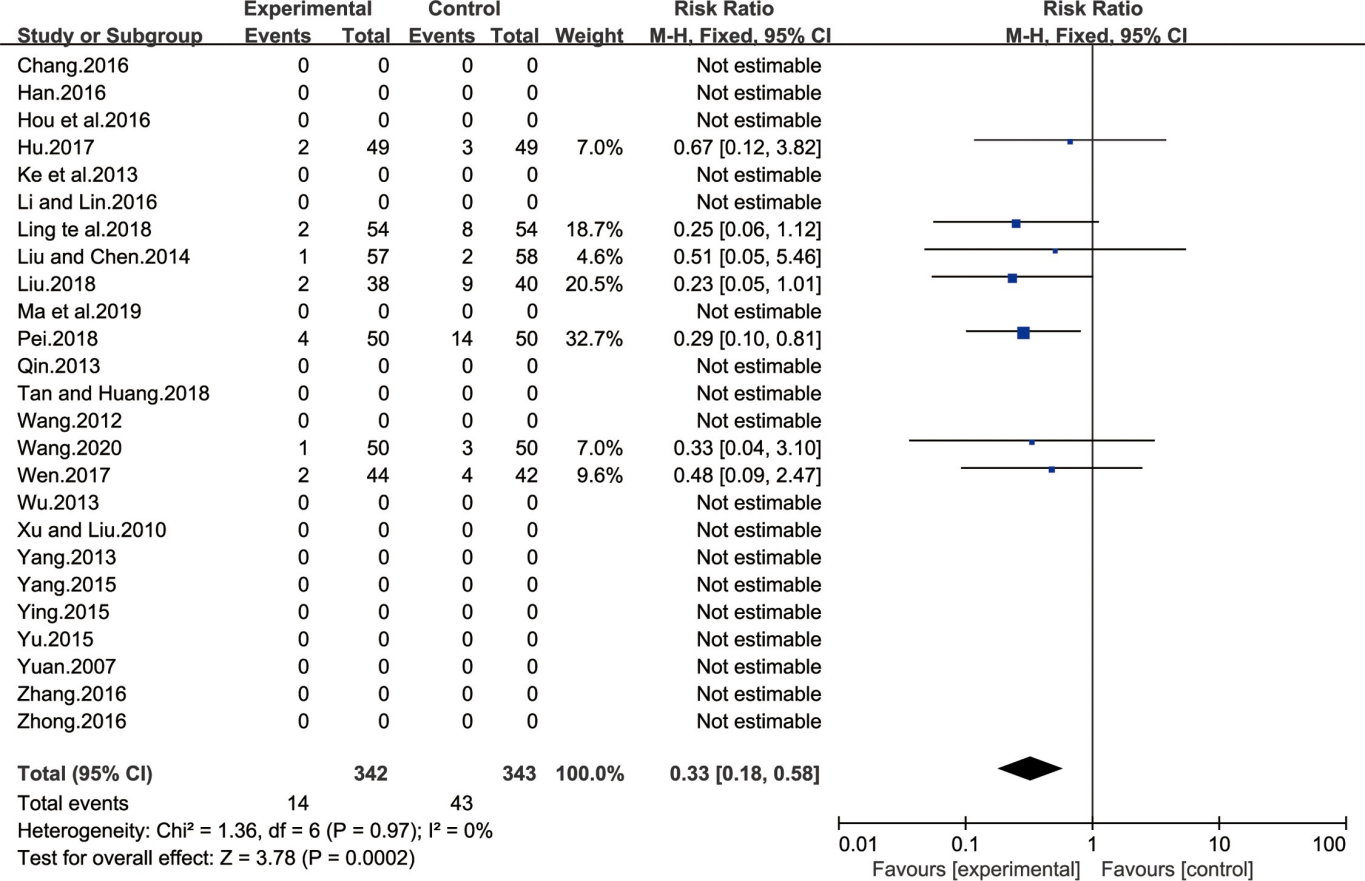
Forest plot of safety evaluation (fixed effect model).

All twenty-six studies were included in safety evaluation. Fifteen of all the studies mentioned whether adverse reactions were observed in the treatment course [13,15–17,21,26–32,34,35,38]. Among the fifteen studies, eight studies mentioned that no adverse reactions were observed in patients [21,26,28–32,34]. Seven studies mentioned the specific number of patients with adverse reactions in each group [13,15–17,27,35,38]. Other studies did not mention adverse reactions. The results showed heterogeneity test were P = 0.97 and I^2^ = 0%, which showed that the study had a homogeneity, so the fixed effect model was adopted for analysis. BXD showed statistically significant differences in safety evaluation compared to the control group (MD 0.33; 95% CI 0.18, 0.58; P = 0.0002). The test for overall effects: Z = 3.78. The results showed that safety evaluation in CAG patients with BXD was better than the control group (Fig 12).

#### Fell off and followed-up cases

Eight of all the studies [27,30–32,34–35,37–38] mentioned whether the cases withdrawn or fell off during the treatment. One case withdrawn from the Ke Xue [30] experiment group; three cases withdrawn from the Liu Haiyan [27] experiment group, two cases withdrawn from the Liu Haiyan [27] control group; seven cases withdrawn from the Liu Maoxian [35] experiment group, five cases withdrawn from the Liu Maoxian [35] control group; one case fell off from the Yuan Chengye [34] experiment group; Four of the studies mentioned cases where there was no withdrawal or dropped out[31–32,37–38]. Other studies did not mention the withdrawal or fell off cases. In addition, none of the twenty-six included studies [13–36,37–38] mentioned followed-up after treatment.

## Discussion

BXD is a classic formula of TCM, which is recorded in an antique medical book of *Shang Han Lun* written by Zhang Zhongjing in Han Destiney. BXD consists of seven traditional Chinese herbs including Ban Xia (Rhizoma Pinelliae), Huang Qin (Radix Scutellariae), Huang Lian (Rhizome Coptidis), Gan Jiang (Rhizoma Zingibers), Gan Cao (Radix Glycyrrhizae), Da Zao (Fructus Ziziphi Jujubae), and Ren Shen (Radix Ginseng). Our study showed that:1) BXD showed better clinical efficacy rate than the control group, especially in improving symptoms of CAG patients; 2) BXD showed more effects in inhibiting HP, improving HP-related inflammation, and relieving the degree of glandular atrophy, IM and dysplasia of GM; 3) BXD was safer than the control group and had fewer adverse events. In addition, by strictly following the TCM theory of syndrome differentiation, dosage and/or formula in a certain decoction can be added or subtracted according to individual's symptom of CAG patients [39]. Hence, BXD has advantages of flexibility in treating CAG.

Many studies [40–60] have shown the efficacy of BXD for CAG patients in reducing inflammation, preventing cancer, and protecting GM, which is consistent with the outcomes of our study. By suppressing acid secretion and promoting gastric emptying, BXD significantly improved symptoms such as stomach distending pain and belching [40]. In addition, evidences [41–43] in modern pharmacological studies have shown that BXD is an effective prescription for reliving the degree of glandular atrophy, IM and dysplasia of GM, whose mechanisms are possibly associated with inhibiting repeated damages caused by HP-related inflammation. Among all the herbs in BXD, Huanglian, Huangqin and Ganjiang can inhibit HP [44–48]. Besides, berberine, a natural isoquinoline alkaloid from Huanglian, possesses therapeutic effects on H. pylori-induced chronic atrophic gastritis [49]. Gancao can reduce injuries of GM [50]. Banxia and Huanglian can protect GM from erosion [51–53]. In a word, by reducing inflammatory response, BXD reversed these morphological alterations of GM in a certain degree [54]. Some studies [55–57] have shown that Renshen and Huanglian can relive pain by inhibiting central nervous system. Also, Ganjiang and Banxia can stop vomiting [51, 58–60]. Therefore, the prescription has effects in preventing the occurrence of adverse events such as nausea, stomachache, and insomnia.

Several limitations of this meta-analysis must be acknowledged. Firstly, the methodological quality of included studies was not better and only one trial mentioned the double blinding. Secondly, the course of disease and treatments was not completely consistent in some included studies. This might potentially compromise the validity of some results and led to optimistic outcomes for treatment. Thirdly, the courses of treatment in the included studies were all less than six months. Considering atrophic gastritis as a chronic recurrent disease, its treatment sessions and follow-up periods should be long enough to evaluate long-term clinical effect of BXD.

## Conclusions

The meta-analysis showed that BXD was more effective and safer for CAG patients than the control group. However, due to limitations of methodological quality and small sample size of the included studies, further standardized research of rigorous design should be needed.

## Supporting information

S1 Checklist PRISMA 2009 checklist. (DOC)

S1 Diagram Flow diagram of included studies. (DOC)

S1 File A sample search strategy. (DOCX)
