# Supplementary material for: Efficacy of Banxia Xiexin decoction for chronic atrophic gastritis: A systematic review and meta-analysis
Source: PLoS One. 2020 Oct 27;15(10):e0241202. doi: 10.1371/journal.pone.0241202 (PMC7591022; doi:10.1371/journal.pone.0241202)
Supplement: S1 Diagram — (DOC) [file pone.0241202.s002.doc]

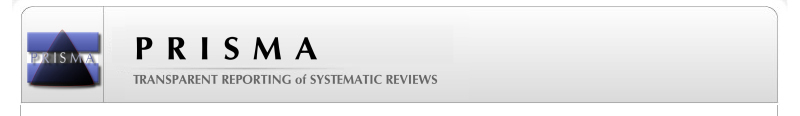
**PRISMA 2009 Flow Diagram**

**Screening**

**Included**

**Eligibility**

**Identification**

Records identified through database searching
(Chinese n=449)

Additional records identified through other sources
(English n =5)

Records after duplicates removed
(n = 449)

Records screened
(n =53)

Records excluded, with reasons
clinical experience report (n=80)

review (n=27)

irrelevant to our topic (n=266)

irrelevant to CAG (n=23)

Full-text articles assessed for eligibility
(n = 26)

Full-text articles excluded, with reasons
Jadad score <2 (n = 27)

Studies included in qualitative synthesis
(n = 26)

Studies included in quantitative synthesis (meta-analysis)
(n = 26)
