# Supplementary material for: Efficacy of Banxia Xiexin decoction for chronic atrophic gastritis: A systematic review and meta-analysis
Source: PLoS One. 2020 Oct 27;15(10):e0241202. doi: 10.1371/journal.pone.0241202 (PMC7591022; doi:10.1371/journal.pone.0241202)
Supplement: S1 File — (DOCX) [file pone.0241202.s003.docx]

Full search strategy and search terms of PubMed：

＃1 Search chronic atrophic gastritis [Me SH Terms]

＃2 Search precancerous lesions of gastric cancer [Title/Abstract]

＃3 Search atrophic gastritis [Title/Abstract]

＃4 Search # 1 OR # 2 OR # 3

＃5 Search banxia xiexin [Title/Abstract]

＃6 Search banxiaxiexin [Title/Abstract]

＃7 Search cold-heat complex syndrome [Title/Abstract]

＃8 Search # 5 OR # 6 OR # 7

＃9 Search randomized controlled trial [Title/Abstract]

＃10 Search # 4 AND # 8

＃11 Search # 4 AND # 8 AND # 9
